# SPCS2 serves as a critical host factor for JEV replication by regulating viral protein stability and virion assembly

**DOI:** 10.1128/spectrum.03848-25

**Published:** 2026-03-30

**Authors:** Bei Niu, Shi-Meng Liu, Shu-Jian Zhang, Yu-Ting Huang, Jian-Hui Zhang, Sen Hu, Zhi-Gao Bu, Rong-Hong Hua

**Affiliations:** 1 State Key Laboratory of Animal Disease Control and Prevention, Harbin Veterinary Research Institute, Chinese Academy of Agricultural Sciences, Harbin, China; 2 Jiangsu Co-innovation Centre for Prevention and Control of Important Animal Infectious Disease and Zoonoses, Yangzhou University, Yangzhou, China; Chinese Academy of Sciences, Wuhan Institute of Virology, Wuhan, China

**Keywords:** viral replication, protein-protein interactions, NS2B, SPCS2, flavivirus, host factor

## Abstract

Non-structural protein 2B (NS2B) of flaviviruses plays multiple versatile roles in viral replication through interacting with viral proteins and cellular factors. Identification of NS2B-interacting host factors is essential for a detailed understanding of the viral life cycle, as well as for the development of antiviral therapeutics. To identify the cellular factors that interact with NS2B and are important for JEV replication, JEV-infected cell lysates were co-immunoprecipitated and analyzed by mass spectrometry. Signal peptidase complex subunit 2 (SPCS2) was identified as a novel host factor that interacts with JEV NS2B. SPCS2 was also found to interact with JEV NS5. We revealed that SPCS2 is a host factor required for the replication of JEV by silencing and knocking out endogenous SPCS2. Knockout of SPCS2 markedly impaired intracellular virion assembly and production of infectious JEV particles. Furthermore, we found that SPCS2 depletion promoted the degradation of viral prM, E, and NS1 proteins, but not NS2B protein. Functional loss of SPCS2 does not influence viral attachment, cell entry, RNA replication, protein translation and processing, or even the formation of endoplasmic reticulum membrane-invaginated vesicles during the life cycle of JEV replication. Our findings suggest that SPCS2, a novel host factor that interacts with NS2B and NS5, plays a crucial role in maintaining viral protein stability and in the assembly of JEV virions.

**IMPORTANCE:**

The flavivirus non-structural protein, NS2B, participates in viral replication and assembly by interacting with viral proteins and host cellular factors. However, the currently known viral replication, which requires host factors that interact with NS2B, is still very limited. In this study, we identified SPCS2 as a novel NS2B-interacting host factor required for JEV replication using co-immunoprecipitation and mass spectrometry analyses. We revealed that SPCS2 plays essential roles in maintaining the stability of the viral proteins prM, E, and NS1, and in the assembly of infectious JEV particles. This study enriches our understanding of the molecular details underlying host factor involvement in the JEV replication life cycle.

## INTRODUCTION

Japanese encephalitis virus (JEV) is a positive-stranded RNA virus belonging to the *Orthoflavivirus* genus in the *Flaviviridae* family. JEV, which causes Japanese encephalitis (JE), is a zoonotic, mosquito-borne viral disease in humans, birds, swine, and horses. Although most JEV infections are asymptomatic in humans, there are still approximately 68,000 clinical cases of JE with approximately 13,600 to 20,400 deaths each year worldwide (1, 2). Although vaccines are available for the prevention of Japanese encephalitis (JE) (3–5), specific therapeutics are lacking. A comprehensive understanding of the replication mechanism of JEV can serve as a critical foundation for the development of specific therapeutic agents. In particular, fully screening the host factors that interact with the virus and participate in viral replication could provide potential antiviral targets (6).

The whole genome of JEV comprises a single open reading frame encoding a large polyprotein that is processed by cellular and viral proteases into three structural and seven non-structural proteins (7). Among the non-structural proteins, NS2B mainly functions as a cofactor for the enzymatic activity of the NS3 proteinase (8). NS2B is an integral membrane protein that contains two transmembrane regions at the N-terminus, a transmembrane region at the C-terminus, and a central hydrophilic outer membrane domain. The outer membrane domain is essential for the activation of NS3 protease function. In addition to acting as a cofactor for NS3 protease, NS2B also plays important roles in viral replication and virion assembly by interacting with viral proteins (9, 10). Additionally, the Flavivirus NS2B protein interacts with cellular host factors to regulate viral replication. JEV NS2B interacts with SPCS1 to regulate the virion assembly of JEV (11). The interaction between JEV NS2B-3 and AXL promotes the release of JEV (12). DENV NS2B was found to interact with MAVS and IKKε to impair RIG-I-directed antiviral response (13). ZIKV NS2B interacts with PP1α to promote ZIKV replication (14). These data indicate that beyond its canonical role as a cofactor of NS3, the flavivirus NS2B protein may play multiple versatile roles in viral replication by interacting with host factors.

The signal peptidase complex (SPC) is an essential membrane complex in the endoplasmic reticulum. The human SPC comprises four subunits: SPCS1, SPCS2, SPCS3, and the catalytic subunit SEC11A or SEC11C (15). The main function of SPC is to remove signal peptides from secretory proproteins. A recent study revealed a new function of human SPC, acting as a quality control enzyme for membrane proteins by cleaving incorrectly folded membrane proteins at cryptic cleavage sites (16). In addition to these physiological functions within cells, SPC plays a significant role in the replication of various viruses. For instance, SPC participates in the cleavage of polyproteins of many flaviviruses (e.g., JEV, WNV, ZIKV, DENV, and HCV) (17) as well as HIV (18). During polyprotein processing, the non-catalytic subunit SPCS1 affects the processing efficiency and the assembly of viral particles by interacting with viral proteins (11, 19, 20). Among the three non-catalytic subunits of SPC, SPCS1 and SPCS3 have been identified as pro-viral host factors for flavivirus (17), but the role of SPCS2 in the flavivirus life cycle remains unknown.

In this study, we identified SPCS2 as a host factor that interacts with JEV NS2B using immunoprecipitation mass spectrometry (IP-MS) analysis. In addition, SPCS2 interacts with JEV NS5. We further revealed that SPCS2 is a host factor required for JEV replication by knocking down and knocking out endogenous SPCS2.

## RESULTS

### SPCS2 interacts with JEV NS2B and NS5 proteins

To identify the host proteins interacting with NS2B in JEV-infected cells, immunoprecipitation was conducted using the JEV NS2B-specific monoclonal antibody (mAb) 14A3 with JEV-infected cell lysates (Fig. S1). The associated proteins were subsequently analyzed using mass spectrometry. After three repeated experiments, SPCS2 consistently appeared in the mass spectrometry results. These results indicated that SPCS2 may interact with NS2B.

**Fig 1:**
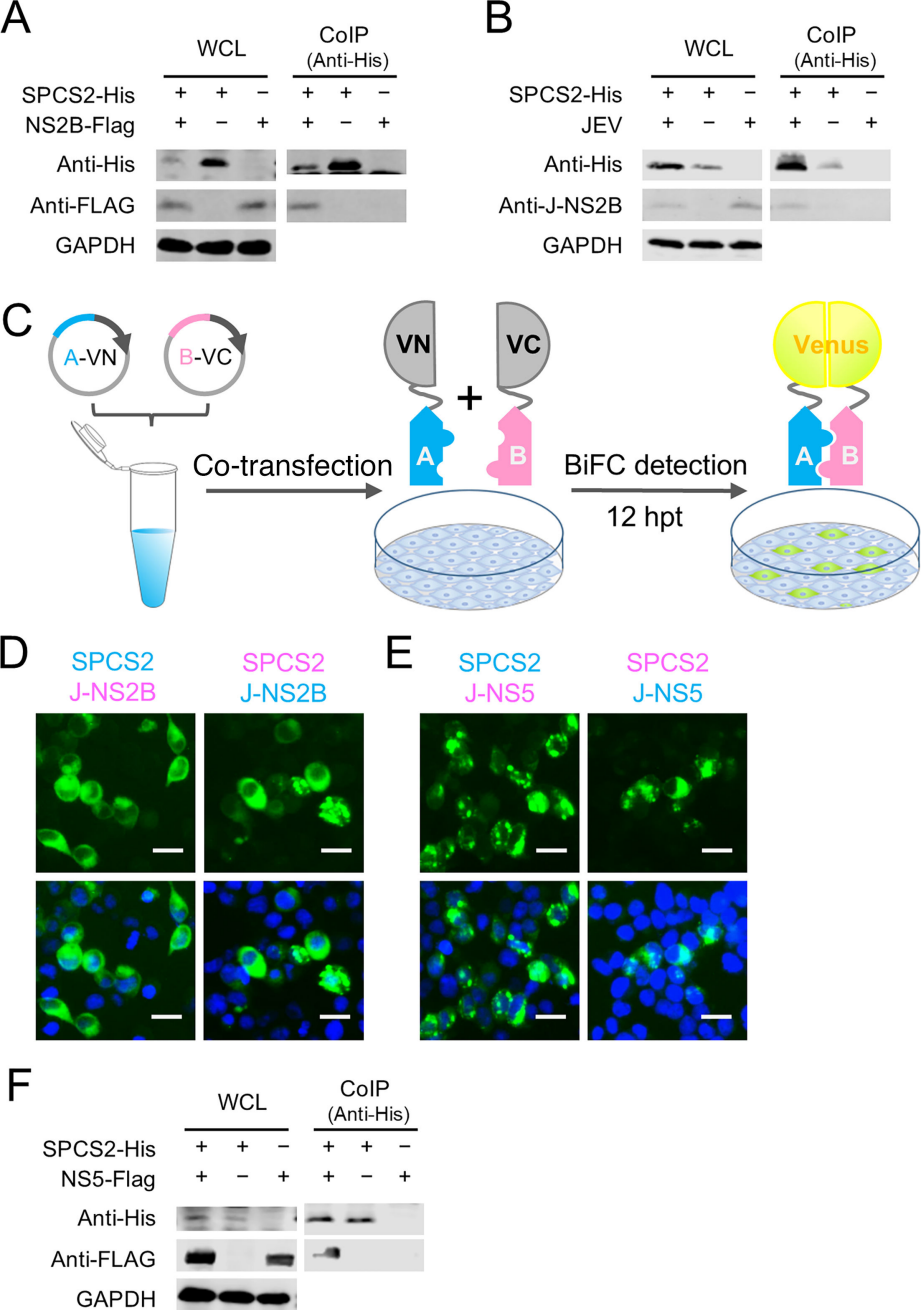
Interaction of SPCS2 with JEV NS2B and NS5 proteins in mammalian cells. (**A**) His-tagged SPCS2 (SPCS2-His) and FLAG-tagged JEV NS2B (NS2B-Flag) were co-expressed in HEK-293T cells. Lysates of transfected cells were immunoprecipitated with an anti-6× His tag antibody. The resulting precipitates and whole-cell lysates (WCL) used for immunoprecipitation were detected by immunoblotting using anti-6× His tag and anti-FLAG antibodies. Data from one experiment of two are shown. CoIP, coimmunoprecipitation. (**B**) HEK-293T cells were transfected with a plasmid expressing SPCS2-His. Cells were infected with JEV at an MOI of 0.5 at 24 h post-transfection. At 48 hpi, the cell lysates were immunoprecipitated with an anti-6× His tag antibody. The resulting precipitates and whole-cell lysates used for immunoprecipitation were detected by immunoblotting using anti-6× His tag and anti-NS2B antibodies, respectively. Data from one of two experiments are shown. (**C**) Schematic diagram of BiFC analysis system. Two eukaryotic expressing plasmids of proteins to be analyzed fused with N-terminal fragment (VN) and C-terminal fragment (VC) of the Venus protein, respectively. Twelve hours after co-transfection of the pair of plasmids, the transfected cells were observed using a fluorescence microscope. (**D**) Detection of the SPCS2-NS2B interaction in transfected cells using BiFC system (scale bar: 20 µm). (**E**) Detection of SPCS2-NS5 interaction in transfected cells using BiFC system. (**F**) His-tagged SPCS2 (SPCS2-His) and FLAG-tagged JEV NS5 (NS5-Flag) were co-expressed in HEK-293T cells. Lysates of transfected cells were immunoprecipitated with an anti-6× His tag antibody. The resulting precipitates and whole-cell lysates (WCL) used for immunoprecipitation were detected by immunoblotting using anti-6× His tag and anti-NS5 antibodies. Data from one experiment of two are shown.

To determine the specific interaction between NS2B and SPCS2 in cells, FLAG-tagged NS2B (NS2B-FLAG) was co-expressed in HEK-293T cells with 6×His-tagged SPCS2 (SPCS2-His), followed by co-immunoprecipitation and immunoblotting. FLAG-tagged NS2B was found to be coimmunoprecipitated with SPCS2 (Fig. 1A). Additionally, the native NS2B protein in cells infected with JEV also coimmunoprecipitated with transfected SPCS2 (Fig. 1B). We further confirmed the interaction between NS2B and SPCS2 using a Venus-based BiFC assay (Fig. 1C). Co-expression of the two pairs of SPCS2-VN vs NS2B-VC and SPCS2-VC vs NS2B-VN all resulted in strong fluorescence signals (Fig. 1D). Furthermore, SPCS2 was found to interact with NS5 of JEV after BiFC screening of SPCS2 versus all 10 JEV genome-encoded proteins fused to the C-terminal residues of the Venus protein (VC) (Fig. S2; Fig. 1E). The interaction between SPCS2 and NS5 was also verified by co-immunoprecipitation (Fig. 1F). FLAG-tagged NS5 was co-immunoprecipitated with co-expressed SPCS2.

### SPCS2 is a crucial host factor required for JEV propagation in cells

**Fig 2:**
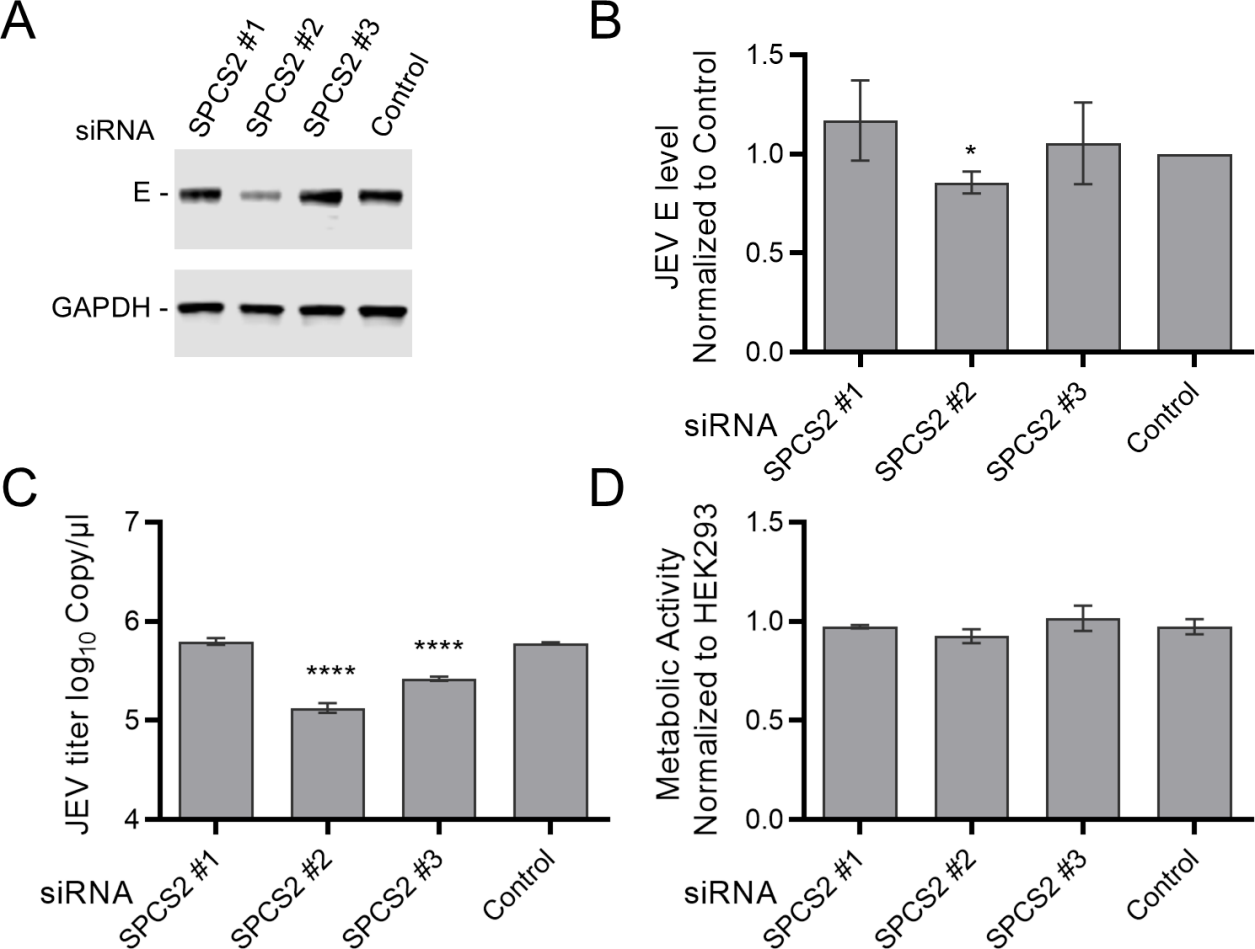
SPCS2 knockdown downregulates the propagation of JEV. HEK-293 cells were transfected with three different siRNAs targeted against SPCS2, or a non-targeting siRNA, at a final concentration of 15 nM. At 48 h post-transfection, the cells were infected with JEV at an MOI of 0.5. Two days after infection, the cell lysates were analyzed by immunoblotting for JEV E protein expression (**A**). Data from one of three experiments are shown. (**B**) E protein expression levels were quantified using band intensities relative to those of GAPDH. Data were normalized to those of non-targeting siRNA control–treated cells. (**C**) Virus titers in the culture supernatants of infected cells were measured using RT-qPCR. (**D**) Cell viability following siRNA transfection was determined using 3-(4,5-dimethylthiazol-2-yl)-2,5-diphenyltetrazolium bromide) tetrazolium (MTT) cell viability assays. Data were pooled from three independent experiments. Values are expressed as the mean ± SD. Statistical significances were determined by one-way ANOVA, compared to non-targeting siRNA treated cells (*, *P* < 0.05; **, *P* < 0.01; ***, *P* < 0.001; ****, *P* < 0.0001).

To investigate the role of SPCS2 in JEV replication, RNA interference assays were performed to silence the endogenous SPCS2 gene. HEK293 cells were transfected with SPCS2-targeted or non-targeted control small interfering RNAs (siRNAs). Twenty-four hours post-transfection, the cells were infected with JEV at an MOI of 0.1. At 48 h post-infection (hpi), the JEV E protein in cell lysates was detected by western blotting using the E protein-specific monoclonal antibody 5E7. Virus titers in the supernatants were determined using RT-qPCR. Among the three SPCS2-specific siRNAs, an obvious reduction in the E protein was observed after treatment with #2-siRNA (Fig. 2A). The E protein quantitative analysis results showed that #2-siRNA significantly reduced E protein levels compared to the control siRNA (Fig. 2B). The RT-qPCR results showed that the virus titers were significantly decreased after treatment with #2-siRNA and #3-siRNA (Fig. 2C). We further confirmed through a cell viability assay that the results were not due to the cytotoxic effects of transfection (Fig. 2D). These results imply that SPCS2 may be a necessary host factor for JEV replication in cells.

**Fig 3:**
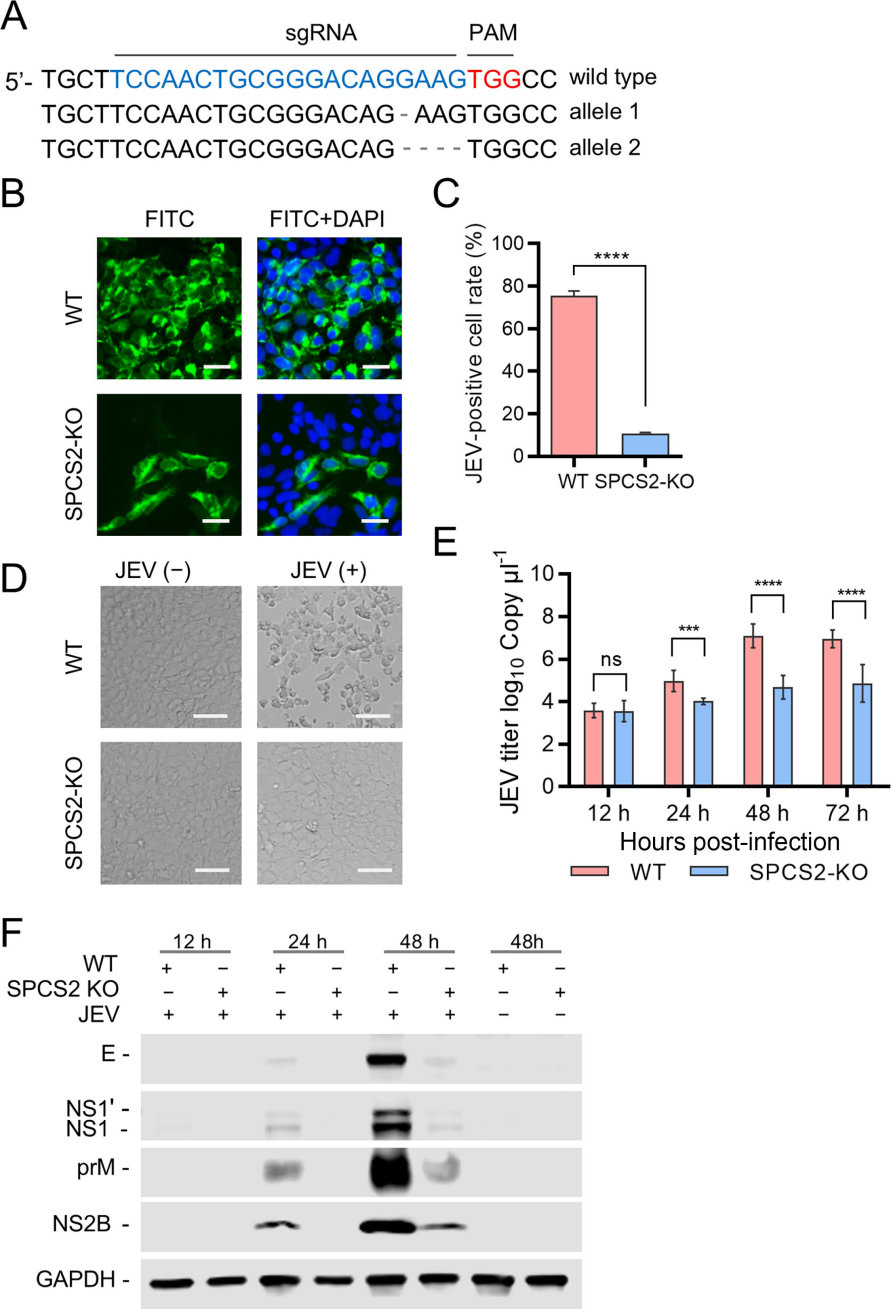
SPCS2 knockout impairs JEV propagation. (**A**) Sequencing of SPCS2 alleles in gene-edited HEK-293 cells after limiting-dilution cloning. The single guide RNA targeting site and protospacer adjacent motif (PAM) sequences are highlighted above the WT gene. Deleted nucleotides are indicated by dashes. (**B**) WT and SPCS2-KO cells were infected with JEV at an MOI of 0.05. At 48 hpi, cells were fixed and probed with JEV E-specific antibody by an immunofluorescence assay (scale bar: 20 µm). Data from one experiment of three are shown. (**C**) The rate of JEV-positive cells was examined using flow cytometry assay. The data are the averages of results from three independent experiments performed in triplicate. Statistical significance was determined using Student’s *t*-test (****, *P* < 0.0001). (**D**) Cytopathic effects were observed at 72 hpi by microscopy (Scale bar: 60 µm). Data from one of three experiments are shown. (**E**) Comparison of viral titers in the supernatants of WT and SPCS2-KO cells infected with JEV at an MOI of 0.05. At 12, 24, 48, and 72 hpi, the titers of JEV in the supernatant of infected cells were examined using RT-qPCR. The data were pooled from three experiments performed in duplicate. Statistical significance was determined by Student’s *t*-test (ns, *P* > 0.05; ***, *P* < 0.001; ****, *P* < 0.0001).(**F**) Expression of the E, NS1, prM, and NS2B proteins in infected cells was analyzed by immunoblotting with indicated antibodies. Data from one experiment of two are shown.

To further elucidate the role of SPCS2 in JEV propagation in cells, we generated an SPCS2 knockout HEK293 cell line (SPCS2-KO) using CRISPR/Cas9 gene editing (Fig. 3A). Wild-type (WT) and SPCS2-KO cells were infected with JEV. At 48 hpi, the cells were fixed and immunofluorescence-stained with E protein-specific mAb 5E7. As shown in Fig. 3B, SPCS2 depletion resulted in a reduction in infection. Flow cytometry assay results indicated that the infectivity of JEV in SPCS-KO cells was significantly lower than that in WT cells (Fig. 3C). Another direct result was that SPCS2 depletion almost completely eliminated the cytopathic effects of JEV infection (Fig. 3D). Viral titers in the supernatant of infected cells at the indicated time points were determined using RT-qPCR. As shown in Fig. 3E, the viral titers in the supernatants of SPCS2-KO cells at 24, 48, and 72 hpi were significantly lower than those in WT cells. Consistently, the expression levels of viral E, prM, and NS1 proteins in SPCS2-KO cells were lower than those in WT cells (Fig. 3F). These results indicate that loss of SPCS2 gene function greatly impairs JEV replication.

**Fig 4:**
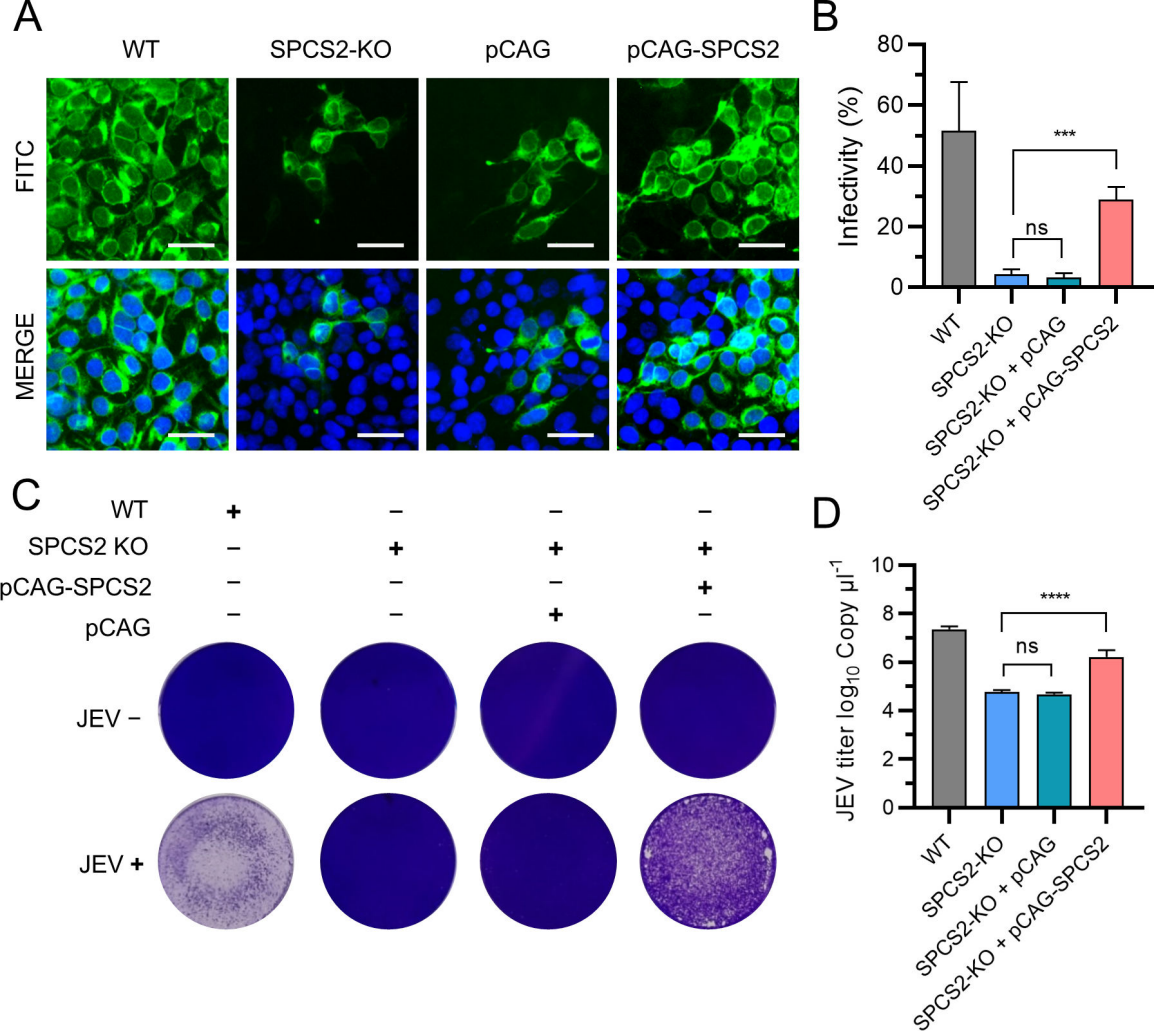
Trans-complementation of SPCS2 rescues the propagation of JEV in SPCS2-KO cells. (**A**) WT and SPCS2-KO cells, SPCS2-KO cells transfected with SPCS2 expressing plasmid or vehicle, were infected with JEV at an MOI of 0.05. At 48 hpi, the cells were fixed and probed with a JEV E protein-specific antibody by IFA. Data from one of three experiments are shown (scale bar: 40 µm). (**B**) Infectivity was analyzed by flow cytometry assay. The data are pooled from three experiments in duplicate. Statistical significances were determined by one-way ANOVA, compared to SPCS2-KO cells (ns, *P* > 0.05; ***, *P* < 0.001). (**C**) Cell cytopathic effects in JEV-infected cells were observed at 72 hpi by a crystal violet staining assay. Data from one of two experiments are shown. (**D**) At 48 hpi, the titers of JEV in the supernatant of infected cells were examined using RT-qPCR. The data are pooled from three experiments in duplicate. Statistical significance was determined by one-way ANOVA, compared to SPCS2-KO cells (ns, *P* > 0.05; ****, *P* < 0.0001).

To verify the specificity of the effect of SPCS2 depletion on JEV replication, we performed a trans-complementation experiment by transfecting pCAG-SPCS2 into SPCS2-KO cells. The results indicated that transfection with pCAG-SPCS2, but not the vehicle control pCAG, rescued JEV infectivity in SPCS2-KO cells (Fig. 4A and B). Compensation of SPCS2 also recovered the cytopathic effects of JEV infection in SPCS2-KO cells (Fig. 4C). Quantitative analysis also revealed that exogenous compensation of SPCS2 significantly increased virus titers in the cell supernatant (Fig. 4D). These results demonstrate that SPCS2 is a necessary host factor for JEV replication in cells.

### SPCS2 is not involved in cell attachment, entry, or RNA replication during the early stage of infection

**Fig 5:**
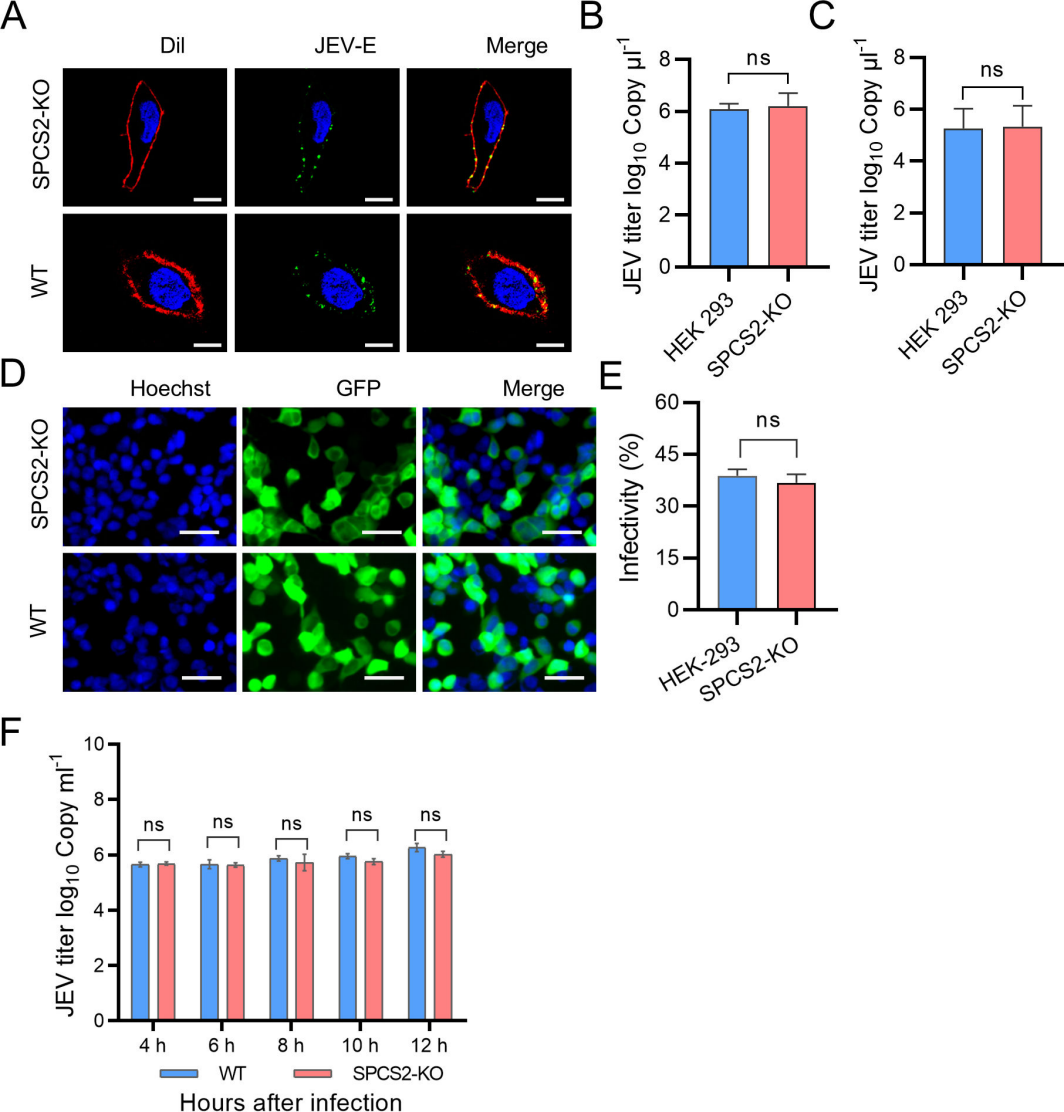
Knockout of SPCS2 does not affect viral attachment, cell entry, or RNA replication in the early stage of infection. (**A**) Viruses attached to WT and SPCS2-KO cells were detected using confocal microscopy. JEV virions were stained with anti-E antibodies. Cell membranes were stained with Dil. Cell nuclei were stained with DAPI. Data from one of two experiments are shown (scale bar: 10 µm). (**B**) Titers of cell-attached viruses were examined using RT-qPCR. The data are pooled from three experiments in duplicate. Statistical significance was determined by Student’s *t*-test (ns, *P* > 0.05). (**C**) After the adsorption and entry processing steps, the viral titers within the cells were determined using RT-qPCR. The data are pooled from three experiments in duplicate. Statistical significance was determined by Student’s *t*-test (ns, *P* > 0.05). (**D**) WT and SPCS2-KO cells were infected with JEV single-round infectious RRPs at an MOI of 0.5. At 48 hpi, the cell nuclei were stained with Hoechst reagent. GFP fluorescence-positive cells indicated RRP infection. Data from one of three experiments are shown (scale bar: 20 µm). (**E**) Infectivity of RRPs in WT and SPCS2-KO cells was analyzed by flow cytometry. The data are pooled from three experiments in duplicate. Statistical significance was determined by Student’s *t*-test (ns, *P* > 0.05). (**F**) WT and SPCS2-KO cells were infected with JEV (strain SA14) at an MOI of 1. At 4, 6, 8, 10, and 12 hpi, the infected cells were harvested, and JEV genome RNAs were analyzed using RT-qPCR. The data are pooled from three experiments in duplicate. Statistical significance was determined by Student’s *t*-test (ns, *P* > 0.05).

To assess the stage of the viral life cycle in which SPCS2 is involved, we first investigated the impact of SPCS2 depletion on viral attachment to the cells. WT and SPCS2-KO cells were incubated with JEV at an MOI of 50 on ice for 1 h. After washing three times with cold PBS, the cells were fixed and subjected to IFA staining with E protein-specific antibody or used to quantify the titers of attached JEV by RT-qPCR. Confocal microscopy revealed that SPCS2 knockout had no impact on viral attachment to cells. JEV attached to the cell surface of SPCS2-KO cells in the same pattern as in WT cells (Fig. 5A). The RT-qPCR results also showed no significant difference in the virus titers attached to the cells of SPCS2-KO and WT cells (Fig. 5B). Next, we examined the impact of SPCS2 depletion on viral entry. The RT-qPCR results indicated no significant difference in the intracellular virus titers of WT and SPCS2-KO cells (Fig. 5C).

To avoid the influence of progeny virus infection on result read-out, we employed a single-round infectious JEV reporter replicon particles (RRPs) (11) to infect SPCS2-KO and WT cells. The RRPs were packaged with the JEV structural protein C-prM-E and a WNV subgenomic replicon that lacked structural proteins and contained a GFP reporter gene (21). The ratio of GFP fluorescence-positive cells was used as a readout for the proportion of virus particles that entered the cells. The results showed that both WT and SPCS2-KO cells could be infected with RRPs (Fig. 5D). The infectivities were comparable, and there were no significant differences in infectivity between the WT and SPCS2-KO cells (Fig. 5E). These results indicate that SPCS2 is not involved in the attachment and entry stages of JEV infection. To assess whether SPCS2 affects JEV genome RNA replication at the early infection stage, viral genome RNA levels in cells were examined by RT-qPCR at 4, 6, 8, 10, and 12 hpi. The results demonstrated that there were no significant differences between WT and SPCS-KO cells at all the indicated time points (Fig. 5F). These data suggest that SPCS2 is not involved in viral genome replication at the early stage of infection.

### SPCS2 does not regulate the translation of viral proteins but affects the stability of E and NS1 proteins

**Fig 6:**
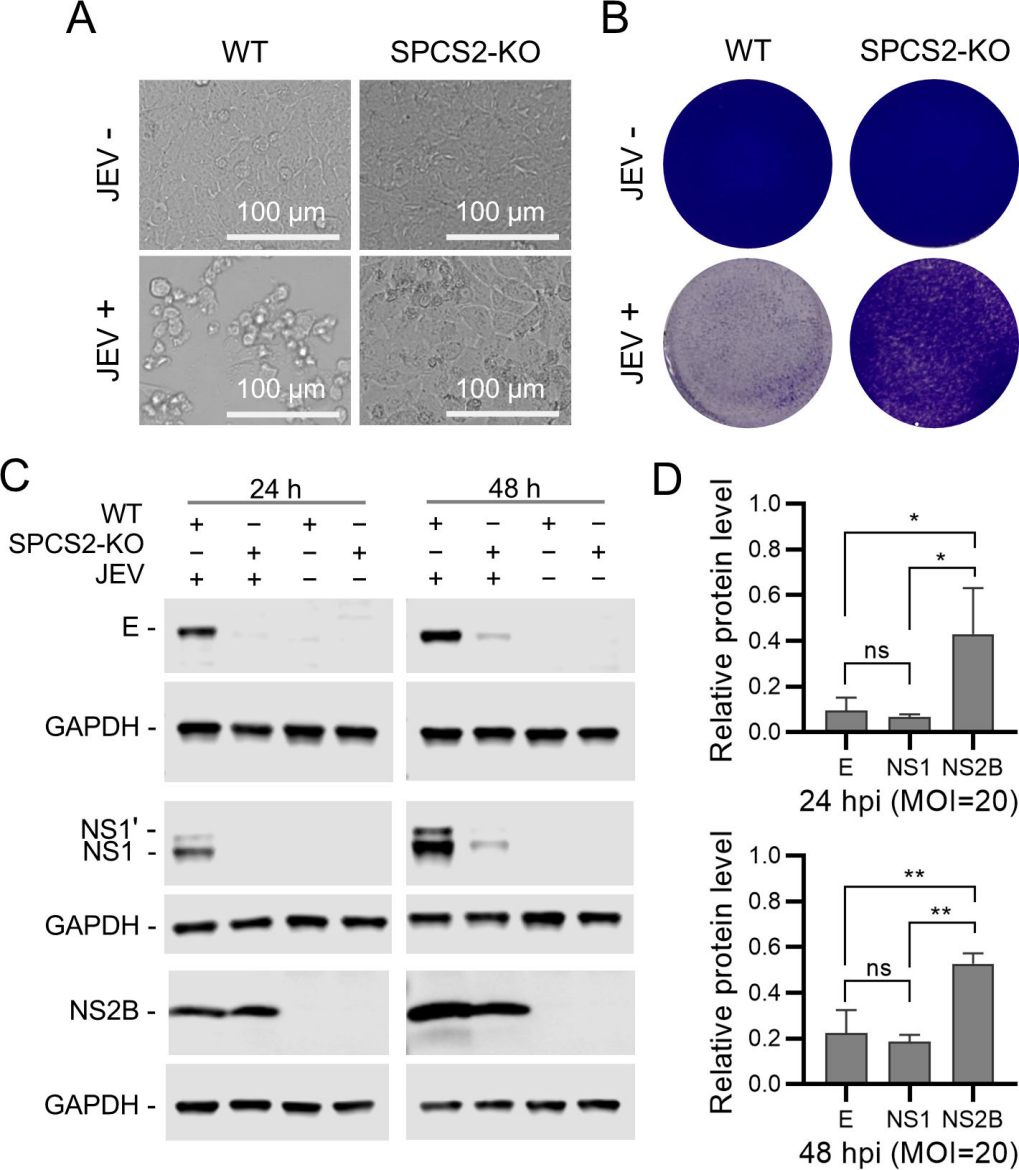
Effect of SPCS2 knockout on JEV protein translation. WT and SPCS2 cells were infected with JEV at an MOI of 20. Cell cytopathic effects were observed using bright-field microscopy (**A**) or by staining with crystal violet (**B**) at 72 hpi. Cell cytopathic data from one of two experiments are shown. At 24 and 48 hpi, respectively, expression of the E, NS1, prM, and NS2B proteins was analyzed by immunoblotting with indicated antibodies (**C**). Data from one of two experiments are shown. (**D**) Protein expression levels were quantified using band intensities relative to those of GAPDH. Data were normalized to those of WT cells. Statistical significance was determined by one-way ANOVA multiple comparisons test (ns, *P* > 0.05; *, *P* < 0.05; **, *P* < 0.01).

To investigate whether SPCS2 is involved in the translation of viral proteins, SPCS2-KO and WT cells were infected with a relatively high dose of JEV at an MOI of 20. It was found that even under high-dose infection, JEV did not cause a cytopathic effect in SPCS2-KO cells (Fig. 6A and B). At 24 and 48 hpi, the viral proteins in the cell lysates were immunoblotted with the indicated antibodies. The results showed that the levels of E and NS1 proteins in SPCS2-KO cells were lower than those in WT cells (Fig. 6C). However, the NS2B protein in SPCS2-KO cells was expressed at a comparable level to that in WT cells (Fig. 6C). The three proteins in SPCS2-KO cells were quantified at relative levels and normalized to the corresponding proteins in WT cells. Statistical analysis showed that there were no significant differences between the relative levels of E and NS1 at 24 and 48 hpi (Fig. 6D), but the relative levels of NS2B were higher than those of E and NS1 (Fig. 6D). The comparable level of NS2B protein expression indicates that the translation of the viral protein is not affected by the deletion of SPCS2. The viral genome encodes a polyprotein that is processed into structural and non-structural proteins during or after translation. NS2B is downstream of E and NS1. Theoretically, the comparable level of NS2B protein expression can be inferred that E and NS1 proteins should also be normally translated. The discrepancies in the immunoblotting results make it reasonable to speculate that the stability of E and NS1 is affected in SPCS2-KO cells after translation.

### Depletion of SPCS2 leads to the degradation of prM, E, and NS1 proteins through an unknown pathway

**Fig 7:**
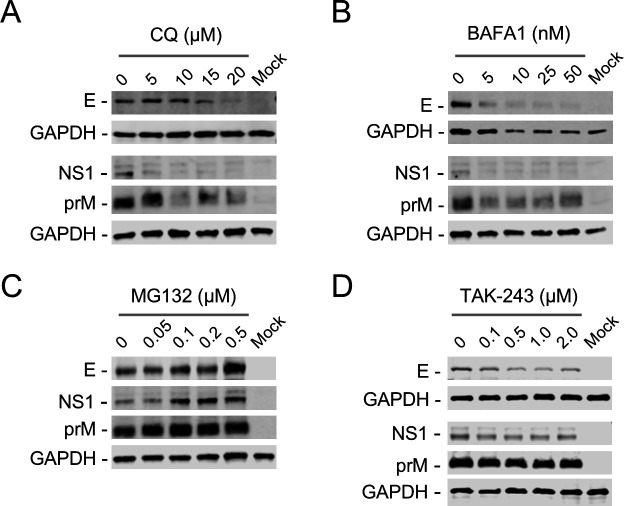
Effects of CQ, BAFA1, MG132, and TAK-243 on the expression of viral proteins in JEV-infected SPCS2-KO cells. SPCS2-KO cells were infected with JEV at an MOI of 20. JEV-infected SPCS2-KO cells were treated with CQ at the indicated concentrations (**A**), BAFA1 at the indicated concentrations (**B**), or MG132 at the indicated concentrations (**C**), or treated with TAK-243 at the indicated concentrations (**D**). Cell lysates were analyzed by western blotting using the indicated antibodies. Blots are representative of three independent experiments.

To survey the mechanism of SPCS2 depletion on the stability of viral proteins, we first used chloroquine (CQ) and bafilomycin A1 (BAFA1), inhibitors of the autophagy lysosome pathway, to treat SPCS2-KO cells during viral infection. Immunoblotting results showed that both CQ and BAFA1 treatments did not recover or even decrease the expression of E, NS1, and prM proteins (Fig. 7A and B). The results indicated that viral protein degradation caused by SPCS2 deletion was independent of the autophagy-lysosomal pathway. We then treated SPCS2-KO cells with the proteasome inhibitor MG132 during viral infection. Immunoblotting results showed that the E, NS1, and prM protein levels increased in a dose-dependent manner (Fig. 7C). We further evaluated the effects of TAK-243, a ubiquitin-activating enzyme (UAE) inhibitor, on viral protein expression in SPCS2-KO cells. Unexpectedly, TAK-243 treatment did not promote the expression of E, NS1, and prM proteins (Fig. 7D). These results suggest that viral proteins are degraded by MG132-sensitive proteases but not through the ubiquitin-proteasome pathway.

### SPCS2 is required for JEV assembly but not for replication organelle formation

**Fig 8:**
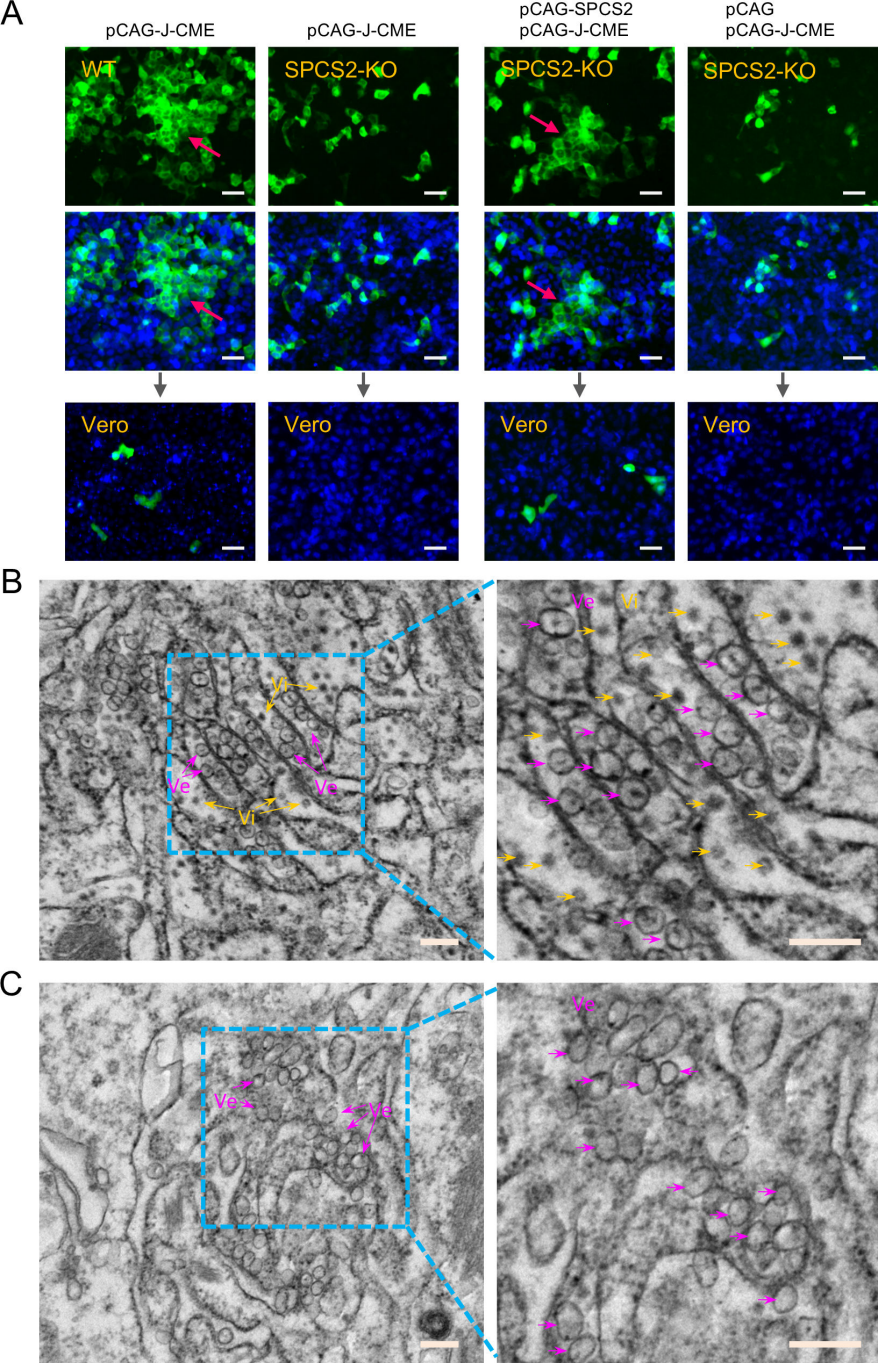
SPCS2 is required for viral assembly of JEV. (**A**) A chimeric JEV reporter replicon particle packaging system was used to survey viral assembly of JEV in SPCS2-KO cells. WT and SPCS2-KO cells were transfected with the indicated plasmids. At 24 h post-transfection, the cells were infected with reporter replicon particles. At 48 hpi, the cells were observed using a fluorescence microscope, and the cell supernatants were harvested to infect Vero cells. The infected Vero cells were observed using a fluorescence microscope at 48 hpi (Scale bar: 40 µm). Viral assembly was also evaluated using transmission electron microscopy. Electron micrographs of WT cells (**B**) and SPCS2-KO cells (**C**) infected with JEV. Virus particles (Vi) are indicated by yellow arrowheads. Viral replication vesicles (Ve) are indicated by purple arrowheads. The right panel shows an enlarged view of the cyan box area in the left panel.

To investigate whether SPCS2 is involved in the assembly or release of JEV virions, we performed chimeric JEV reporter replicon particle (RRP) packaging experiments in SPCS2-KO cells. This packaging system was based on single-round infectious JEV reporter replicon particles and the JEV structural protein-expressing plasmid pCAG-J-CME (11, 21). After transfection with pCAG-J-CME and infection with RRPs in WT HEK293 cells, in addition to sporadic GFP-positive cells, there were GFP-positive cells clustered into fluorescence foci (Fig. 8A, first panel from the left). The formation of fluorescence foci indicated that adjacent cells were infected by newly produced RRPs. Furthermore, GFP-positive cells are present in the supernatant-infected Vero cells (Fig. 8A, first panel from the left). GFP-positive cells indicated the production of infectious JEV RRPs. However, no GFP foci were observed in SPCS2-KO cells, and only sporadic GFP-positive cells were observed. Inoculation of Vero cells with the supernatant resulted in no GFP-positive cells (Fig. 8A. Second panel from the left). These results demonstrate that SPCS2 deletion impairs the assembly of JEV RRPs. To further confirm the function of SPCS2 in virion assembly, we compensated for SPCS2 function in the RRPs packaging experiment by cotransfecting pCAG-J-CME and pCAG-SPCS2. As shown in Fig. 8A (third panel from the left), the introduction of exogenous SPCS2 into SPCS2-KO cells restored the formation of GFP foci and the production of infectious RRPs progeny in the supernatant. As a negative control, the vehicle pCAG did not recover the formation of GFP foci and production of infectious RRPs in the supernatant (Fig. 8A, fourth panel from the left).

We further investigated the effects of SPCS2 depletion on virion assembly using transmission electron microscopy (TEM). WT and SPCS2-KO cells were infected with JEV at an MOI of 20. At 48 hpi, the infected cells were processed for TEM analysis. In the WT cells, abundant progeny virions were observed (Fig. 8B). However, viral progeny production was significantly attenuated in the SPCS2-KO cells. Nearly no typical virion-like structures were observed in the endoplasmic reticulum of SPCS2-KO cells (Fig. 8C). Although viral progeny production was attenuated, viral replication vesicles were evident in SPCS2-KO cells (Fig. 8C). Viral replication vesicles are characteristic organelles of orthoflavivirus replication. These results indicate that SPCS2 is essential for JEV virion assembly but not for the formation of replication organelles.

## DISCUSSION

Cellular factors participate in all facets of viral replication cycle through virus-host interactions. Identification of host factors required for viral replication is essential for our full understanding of viral replication and for the development of novel antiviral therapeutics. Genome-wide silencing and CRISPR screening approaches have been adopted to identify host factors required for flavivirus replication (17, 22–28). The advantage of this screening approach is that it can screen host factors that are important for viral replication on a genome-wide scale; however, it is inferior in screening host factors that interact with specific viral proteins. In order to fully understand the role and mechanism of the NS2B protein in flavivirus replication, we screened host factors that interact with JEV NS2B using co-immunoprecipitation and mass spectrometry analysis. Using this approach, we identified SPCS2 and more than 10 other host proteins that were repeatedly detected in the mass spectrometry results. Among these hits, subunits of endoplasmic reticulum membrane complex (EMC1 and EMC3) and ARF4 have been previously identified as host factors required for flavivirus replication (6, 17, 22–25, 29, 30). These data confirm the validity and reproducibility of our screening approach. However, the interactions between these factors and NS2B require future verification. We previously demonstrated that SPCS1 is a critical host factor required for JEV replication and interacts with NS2B (11). To determine whether NS2B interacts directly with SPCS2, rather than indirectly via its association with SPCS1, co-immunoprecipitation assays were performed not only in JEV-infected cells but also in cells co-expressing FLAG-tagged NS2B and His-tagged SPCS2. Additionally, bimolecular fluorescence complementation (BiFC) assays were conducted in SPCS1 knockout cells to further validate this direct interaction (data not shown). Our findings indicate that JEV NS2B interacts with SPCS1 and SPCS2 within the SPC complex. However, the molecular details underlying the interaction with SPCS2 remain to be elucidated.

The signal peptidase complex subunit 2 is a component of the signal peptidase complex that canonically functions in cleaving and removing the signal peptide from secretory and membrane proteins in the endoplasmic reticulum lumen. Within the complex, SPCS2 interacts with SPCS1 and the catalytic subunit (SEC11A or SEC11C) through the cytosolic domain and the hydrophobic transmembrane regions, respectively (15). As a non-catalytic subunit, SPCS2 does not directly process signal peptides. SPCS2 is associated with newly synthesized tail-anchored and signal-anchored membrane proteins, suggesting that SPCS2 may play an important role in the membrane insertion of membrane proteins (31). In yeast cells, Spc2 is involved in substrate recognition and cleavage site identification (32). However, the function of SPCS2 in mammalian cells remains poorly understood. In particular, the role and function of this factor in viral replication have received relatively few reports. A comprehensive interactome study using tandem affinity purification and proximity-labeling strategies identified that SPCS2 interacts with the S protein of SARS-CoV-2. The interaction between the S protein and SPCS2 was validated using pulldown and western blot assays (33). However, the specific role and molecular mechanism of SPCS2 in the viral life cycle of SARS-CoV-2 was not revealed. An early genome-wide small interfering RNA (siRNA) screen identified SPCS2 as essential for HCV replication in cells. Another recent study showed that SPCS2 interacts with the E2 and p7 proteins of HCV (34). However, the detailed mechanism of SPCS2 in HCV replication has not been elucidated. In this study, we identified for the first time that the NS2B-interacting host factor SPCS2 is required for JEV replication in cells. However, whether SPCS2 is a pan-flavivirus host factor remains to be investigated.

The signal peptidase complex is involved in flavivirus replication. However, the requirement for signal peptidase complex subunits varies at each stage of the flavivirus life cycle. We investigated the stages of SPCS2 involved in the JEV life cycle. For SPCS2 located on the membrane of the endoplasmic reticulum, it is not possible to be involved in viral attachment and entry procedure. As expected, SPCS2 depletion had no effect on cell attachment and entry of JEV. SPCS2 depletion did not affect RNA replication, protein translation, or polyprotein processing. Furthermore, the formation of the characteristic vesicle structure of the virus replication organelles was not affected by the absence of SPCS2. This outcome can be explained by the fact that the translation and processing of viral proteins were not affected by the absence of SPCS2. This result indicates that SPCS2 regulates the replication of JEV through a mechanism distinguished from that of SPCS1. In virus-infected cells or sub-genomic replicons containing cells, the expression of the non-structural proteins of flavivirus is sufficient to induce the formation of the vesicle structure that constitutes the viral replication factory (35). In addition to viral factors, vesicle formation requires the participation of host factors. For instance, receptor for activated protein C kinase 1 (RACK1) plays a crucial role in the formation of viral replication organelles in flavivirus infection. Depletion of RACK1 significantly reduced the formation of vesicle packets in cells (26). Our results indicate that SPCS2 plays a crucial role in maintaining the stability of viral proteins. These proteins include the structural proteins prM and E, as well as the non-structural protein NS1. A common feature of all these proteins is that they enter the endoplasmic reticulum after translation. In the absence of SPCS2, these proteins undergo degradation. However, the specific pathway through which this degradation occurs remains to be determined. It remains to be determined whether SPCS2 directly participates in the protein degradation pathway or plays a role in maintaining the correct folding of proteins within the endoplasmic reticulum. Another function of SPCS2 is to influence the assembly of JEV virion. When infected with a relatively high dose, properly processed viral structural proteins exist, but no viral particle structures are observed in the endoplasmic reticulum. We also used a single-round infectious JEV reporter replicon particle packaging system to verify the role of SPCS2 in the assembly of JEV viral particles.

In summary, using an immunoprecipitation and mass spectrometry screening approach, we identified SPCS2 as a host factor required for JEV replication. SPCS2 interacts with the NS2B and NS5 proteins of JEV. SPCS2 depletion significantly attenuated JEV infection in cells. SPCS2 regulates JEV replication at the virion assembly stage and plays an essential role in maintaining the stability of viral proteins in the endoplasmic reticulum lumen. This study advances our knowledge of the molecular details of flavivirus replication and provides potential targets for the development of antiviral therapeutics.

## MATERIALS AND METHODS

### Cells, viruses, and antibodies

HEK-293 cells (ATCC CRL-1573), HEK-293T cells (ATCC CRL-3216), SPCS2-KO cells (generated from HEK-293 cells as parental cells in this study), and BHK-21 cells (ATCC CCL-10) were cultured in Dulbecco’s modified Eagle’s medium (DMEM) (Sigma-Aldrich; D6429) supplemented with 10% fetal bovine serum (FBS; Gibco). For HEK-293, HEK-293T, and SPCS2-KO cells, cell culture plates were covered with poly D-lysine hydrobromide (Sigma; P7886) at a concentration of 50 μg/mL. BJEV-CME cells (derived from BHK-21 cells stably expressing the JEV C-prM-E protein) (11) were maintained in DMEM supplemented with 10% FBS and 1 mg/mL G418. The JEV SA14 strain was propagated and titrated in BHK-21 cells. A mouse monoclonal antibody against JEV NS2B was generated in this study (Fig. S3). Mouse monoclonal antibodies against the JEV prM, E, and NS1 proteins were generated as previously described (36–38). Other commercial antibodies are listed as follows: Mouse anti-6×His tag antibody (GeneTex, GT359), Rabbit anti-FLAG tag antibody (Proteintech, 20543-1-AP), Rabbit anti-GAPDH antibody (GeneTex, GTX100118), Alexa Fluor 680 donkey anti-mouse IgG (Thermo Life, A10038), IRDye 800CW donkey anti-rabbit IgG (LI-COR, 926-32213), and FITC-labeled goat anti-mouse IgG (ZSGB, ZF-0312).

### Plasmids

All plasmids were constructed using standard procedures and protocols. The plasmids used in this study are listed in the supplemented material (Table S1).

### Western blot

Protein samples were separated by SDS-PAGE and then transferred onto a nitrocellulose (NC) membrane. The membranes were blocked with 4% skim milk and then incubated with a specific primary antibody overnight at 4°C or at room temperature for 2 h. After incubation with primary antibodies at 4°C overnight, the membranes were incubated with Alexa Fluor 680-conjugated or IRDye 800CW-conjugated secondary antibodies for 1 h at room temperature. After washing five times, immunoblot signals were visualized using a near-infrared fluorescence scanning imaging system (Odyssen; Licor). The antibodies used in this study are listed in Table S1.

### Immunoprecipitation

After washing three times with PBS, the transfected and/or infected cells were lysed with RIPA buffer (Sigma, R0278) supplemented with protease inhibitors (Sigma, P8340) and nuclease (Thermo Fisher, 88701) for 30 min on ice. After centrifugation at 12,000 × *g* at 4°C for 10 min, the supernatant was incubated with mouse IgG-conjugated agarose beads (Sigma, A0919) to remove non-specific binding proteins. After incubation for 4 h at 4°C, agarose beads were removed by centrifugation at 6,000 × *g*. The supernatant was incubated with NS2B, NS5, and myc- or FLAG-tag-specific antibodies overnight at 4°C. Protein A/G-conjugated magnetic beads (MCE, HY-K0202) were then added. The incubated beads were washed with washing buffer. Immunoprecipitated proteins were analyzed by SDS-PAGE and western blotting.

### BiFC

BiFC assay was performed as previously described (10, 11). Briefly, to be tested, protein pairs encoding genes were cloned into the VN-tagged plasmid pCAG-VN and VC-tagged plasmid pCAG-VC, or vice versa. Recombinant plasmids were cotransfected into HEK-293T cells. The JEV-NS1-VN and JEV-NS2B-VC pairs were used as negative controls. The pair JEV NS2B-VN and SPCS1-VC was set as a positive control. Twelve hours after transfection, cell nuclei were stained with Hoechst 33,342, and the cells were visualized and photographed using a fluorescence microscope (Life, Evos m5000). The fluorescence-positive ratio of cells was calculated using the ImageJ software.

### RNA interference

HEK293 cells were transfected with 30 nM siRNA using Lipofectamine RNAiMAX (Invitrogen, 13778075). The sequences of siRNA target to SPCS2 were as follows: SPCS2#1: CAGUACUUUAUAUAGUAUATT, SPCS2#2: GUACCUAGCAGUACUUUAUTT, SPCS2#3: CCUUGAUCUGCUGAUUGCATT. The transfected cells were infected with JEV at an MOI of 0.1 at 24 h post-transfection. At 48 hpi, the supernatants were harvested and subjected to virus titration using RT-qPCR. The cells were lysed and subjected to immunoblotting analysis using a JEV E protein-specific antibody.

### Generation of SPCS2 knockout cell lines

SPCS2 knockout cell lines were generated using a CRISPR-Cas9 gene-editing assay. The sgRNA sequence targeting SPCS2 was TCCAACTGCGGGACAGGAAG. First, 20 nt oligos were synthesized and annealed prior to insertion into BbsI-digested plasmid pSpCas9-BB-2A-GFP (PX458) (Addgene, #48138). The plasmid constructs were verified by DNA sequencing. HEK-293 cells were transfected with plasmid DNA using Lipofectamine LTX and PLUS Reagent (Invitrogen, 15338100). Two days later, GFP-positive cells were sorted and single-cloned into 96-cell plates using a flow cytometer. SPCS2 knockout cells were verified by DNA sequencing.

### RT-qPCR

Total RNA was extracted from JEV-infected or uninfected cells with an RNeasy Plus Mini Kit (QIAGEN; 74,104) according to the manufacturer’s instructions. RNA was reverse transcribed into cDNA using the PrimeScript RT Reagent Kit (Takara; RR047A). Real-time PCR was performed as previously described (11). The real-time PCR primers were 5′-AGGGGTTTTCAACTCCATAG-3′ and 5′-CATTAGCCCTTGTGTGATCC-3′. The TaqMan probe was 5′-FAM-TTCTGAAGGCACCACCAAACAC-Eclipse-3′. For JEV viral genome quantification, the plasmid pMD18T-J-E (harboring the JEV E gene) was used as a standard for quantification of JEV genome copy number.

### Immunofluorescence assay

Cells were fixed with 4% paraformaldehyde and then permeabilized with Triton X-100 (for observing viral attachment with confocal microscopy, cells were unpermeabilized). After blocking with BSA, the cells were incubated with JEV E protein-specific mAb 5E7, followed by FITC-conjugated goat anti-mouse IgG. The cell nuclei were stained with DAPI. Cells were observed using a fluorescence microscope (Life, EVOS M5000) or a confocal microscope (ZEISS, LSM980).

### Flow cytometry analysis

HEK-293 and gene-edited cells were infected with JEV at an MOI of 0.1. After 48 h, the cells were dispersed with trypsin. Trypsin in the dispersed cells was inactivated with DMEM supplemented with 10% FBS. After twice washing with PBS, the cells were fixed with 4% paraformaldehyde and then permeabilized with Triton X-100. After blocking with BSA, the cells were incubated with JEV E protein-specific mAb 5E7, followed by FITC-conjugated goat anti-mouse IgG. The cell nuclei were then stained with DAPI. After washing three times with PBS, the cells were processed on a flow cytometer (Apogee, UK), and data were analyzed using FlowJo software.

### Plaque assay

BHK-21 cells were seeded in 24-well plates. Twenty-four hours later, confluent monolayers of BHK-21 cells were infected with serially diluted virus samples. After 6 h, an equal volume of 3% methylcellulose (Sigma, M6385) as the medium in the cell well was added to each well. Three days post-infection, the medium and methylcellulose mixtures were discarded, and the cells were stained with crystal violet-formaldehyde solution. The resulting plaques were counted, and virus titers were calculated.

### Electron microscopy

Cell structures and viral particles were examined using transmission electron microscopy, as previously described (39). Briefly, wild-type HEK293 and SPCS2-KO HEK-293 cells were infected with JEV at an MOI of 1. At 48 h post-infection, the cells were harvested using a cell scraper and centrifuged at 500 × *g* for 5 min. The cell pellets were fixed with 2.5% glutaraldehyde and 0.1% osmium tetroxide, dehydrated with acetone, and embedded in epoxy resin. The embedded cells were sectioned with a Leica EM UC-6 microtome and stained with uranyl acetate and lead citrate. Finally, the sections were observed under an electron microscope (Hitachi, H7650).

## Data Availability

The data underlying this article are available in this article and its Supplement material.

## SUPPLEMENTAL MATERIAL

Supplemental material spectrum.03848-25-s0001.docx Fig. S1 to S3; Table S1.

ASM does not own the copyrights to Supplemental Material that may be linked to, or accessed through, an article. The authors have granted ASM a non-exclusive, world-wide license to publish the Supplemental Material files. Please contact the corresponding author directly for reuse.

## References

[1] Campbell GL, Hills SL, Fischer M, Jacobson JA, Hoke CH, Hombach JM, Marfin AA, Solomon T, Tsai TF, Tsu VD, Ginsburg AS. 2011. Estimated global incidence of Japanese Encephalitis: a systematic review. Bull World Health Org 89:766–774, doi:10.2471/BLT.10.085233 22084515 PMC3209971

[2] Li F, Feng Y, Wang G, Zhang W, Fu S, Wang Z, Yin Q, Nie K, Yan J, Deng X, He Y, Liang L, Xu S, Wang Z, Liang G, Wang H. 2023. Tracing the spatiotemporal phylodynamics of Japanese Encephalitis Virus genotype I throughout Asia and the western Pacific. PLoS Negl Trop Dis 17:e0011192. doi:10.1371/journal.pntd.0011192 37053286 PMC10128984

[3] Duggan ST, Plosker GL. 2009. Japanese Encephalitis Vaccine (inactivated, adsorbed) [IXIARO]. Drugs (Abingdon Engl) 69:115–122. doi:10.2165/00003495-200969010-00008 19192940

[4] Song B-H, Yun G-N, Kim J-K, Yun S-I, Lee Y-M. 2012. Biological and genetic properties of SA₁₄-14-2, a live-attenuated Japanese Encephalitis Vaccine that is currently available for humans. J Microbiol 50:698–706. doi:10.1007/s12275-012-2336-6 22923123

[5] Halstead SB, Thomas SJ. 2011. New Japanese Encephalitis Vaccines: alternatives to production in mouse brain. Expert Rev Vaccines 10:355–364. doi:10.1586/erv.11.7 21434803

[6] Li M-Y, Deng K, Cheng X-H, Siu LY-L, Gao Z-R, Naik TS, Stancheva VG, Cheung PP-H, Teo Q-W, van Leur SW, Wong H-H, Lan Y, Lam TT-Y, Sun M-X, Zhang N-N, Zhang Y, Cao T-S, Yang F, Deng Y-Q, Sanyal S, Qin C-F. 2025. ARF4-mediated intracellular transport as a broad-spectrum antiviral target. Nat Microbiol 10:710–723. doi:10.1038/s41564-025-01940-w 39972062

[7] Chambers TJ, Hahn CS, Galler R, Rice CM. 1990. Flavivirus genome organization, expression, and replication. Annu Rev Microbiol 44:649–688. doi:10.1146/annurev.mi.44.100190.003245 2174669

[8] Falgout B, Pethel M, Zhang YM, Lai CJ. 1991. Both nonstructural proteins NS2B and NS3 are required for the proteolytic processing of dengue virus nonstructural proteins. J Virol 65:2467–2475. doi:10.1128/JVI.65.5.2467-2475.1991 2016768 PMC240601

[9] Yu L, Takeda K, Markoff L. 2013. Protein-protein interactions among West Nile non-structural proteins and transmembrane complex formation in mammalian cells. Virology (Auckl) 446:365–377. doi:10.1016/j.virol.2013.08.006 24074601

[10] Li X-D, Deng C-L, Ye H-Q, Zhang H-L, Zhang Q-Y, Chen D-D, Zhang P-T, Shi P-Y, Yuan Z-M, Zhang B. 2016. Transmembrane domains of NS2B contribute to both viral RNA replication and particle formation in Japanese Encephalitis Virus. J Virol 90:5735–5749. doi:10.1128/JVI.00340-16 27053551 PMC4886793

[11] Ma L, Li F, Zhang J-W, Li W, Zhao D-M, Wang H, Hua R-H, Bu Z-G. 2018. Host factor SPCS1 regulates the replication of Japanese Encephalitis Virus through interactions with transmembrane domains of NS2B. J Virol 92:e00197-18. doi:10.1128/JVI.00197-18 29593046 PMC5974503

[12] Xie S, Liang Z, Yang X, Pan J, Yu D, Li T, Cao R. 2021. Japanese encephalitis Virus NS2B-3 protein complex promotes cell apoptosis and viral particle release by down-regulating the expression of AXL. Virol Sin 36:1503–1519. doi:10.1007/s12250-021-00442-3 34487337 PMC8692519

[13] Nie Y, Deng D, Mou L, Long Q, Chen J, Wu J. 2023. Dengue virus 2 NS2B targets MAVS and IKKε to evade the antiviral innate immune response. J Microbiol Biotechnol 33:600–606. doi:10.4014/jmb.2210.10006 36788451 PMC10236164

[14] Wu X, Zhang L, Liu C, Cheng Q, Zhao W, Chen P, Qin Y, Chen M. 2024. The NS2B-PP1α-eIF2α axis: inhibiting stress granule formation and boosting Zika virus replication. PLoS Pathog 20:e1012355. doi:10.1371/journal.ppat.1012355 38935808 PMC11236161

[15] Liaci AM, Steigenberger B, Telles de Souza PC, Tamara S, Gröllers-Mulderij M, Ogrissek P, Marrink SJ, Scheltema RA, Förster F. 2021. Structure of the human signal peptidase complex reveals the determinants for signal peptide cleavage. Mol Cell 81:3934–3948. doi:10.1016/j.molcel.2021.07.031 34388369

[16] Zanotti A, Coelho JPL, Kaylani D, Singh G, Tauber M, Hitzenberger M, Avci D, Zacharias M, Russell RB, Lemberg MK, Feige MJ. 2022. The human signal peptidase complex acts as a quality control enzyme for membrane proteins. Science 378:996–1000. doi:10.1126/science.abo5672 36454823

[17] Zhang R, Miner JJ, Gorman MJ, Rausch K, Ramage H, White JP, Zuiani A, Zhang P, Fernandez E, Zhang Q, Dowd KA, Pierson TC, Cherry S, Diamond MS. 2016. A CRISPR screen defines a signal peptide processing pathway required by flaviviruses. Nature 535:164–168. doi:10.1038/nature18625 27383988 PMC4945490

[18] Snapp EL, McCaul N, Quandte M, Cabartova Z, Bontjer I, Källgren C, Nilsson I, Land A, von Heijne G, Sanders RW, Braakman I. 2017. Structure and topology around the cleavage site regulate post-translational cleavage of the HIV-1 gp160 signal peptide. eLife 6:e26067. doi:10.7554/eLife.26067 28753126 PMC5577925

[19] Suzuki R, Matsuda M, Watashi K, Aizaki H, Matsuura Y, Wakita T, Suzuki T. 2013. Signal peptidase complex subunit 1 participates in the assembly of hepatitis C virus through an interaction with E2 and NS2. PLoS Pathog 9:e1003589. doi:10.1371/journal.ppat.1003589 24009510 PMC3757040

[20] Alzahrani N, Wu M-J, Sousa CF, Kalinina OV, Welsch C, Yi M. 2022. SPCS1-dependent E2-p7 processing determines HCV Assembly efficiency. PLoS Pathog 18:e1010310. doi:10.1371/journal.ppat.1010310 35130329 PMC8853643

[21] Li W, Ma L, Guo L-P, Wang X-L, Zhang J-W, Bu Z-G, Hua R-H. 2017. West Nile virus infectious replicon particles generated using a packaging-restricted cell line is a safe reporter system. Sci Rep 7:3286. doi:10.1038/s41598-017-03670-4 28607390 PMC5468312

[22] Marceau CD, Puschnik AS, Majzoub K, Ooi YS, Brewer SM, Fuchs G, Swaminathan K, Mata MA, Elias JE, Sarnow P, Carette JE. 2016. Genetic dissection of flaviviridae host factors through genome-scale CRISPR screens. Nature 535:159–163. doi:10.1038/nature18631 27383987 PMC4964798

[23] Savidis G, McDougall WM, Meraner P, Perreira JM, Portmann JM, Trincucci G, John SP, Aker AM, Renzette N, Robbins DR, Guo Z, Green S, Kowalik TF, Brass AL. 2016. Identification of Zika virus and dengue virus dependency factors using functional genomics. Cell Rep 16:232–246. doi:10.1016/j.celrep.2016.06.028 27342126

[24] Li Y, Muffat J, Omer Javed A, Keys HR, Lungjangwa T, Bosch I, Khan M, Virgilio MC, Gehrke L, Sabatini DM, Jaenisch R. 2019. Genome-wide CRISPR screen for Zika virus resistance in human neural cells. Proc Natl Acad Sci USA 116:9527–9532. doi:10.1073/pnas.1900867116 31019072 PMC6510995

[25] Zhao C, Liu H, Xiao T, Wang Z, Nie X, Li X, Qian P, Qin L, Han X, Zhang J, Ruan J, Zhu M, Miao Y-L, Zuo B, Yang K, Xie S, Zhao S. 2020. CRISPR screening of porcine sgRNA library identifies host factors associated with Japanese Encephalitis Virus replication. Nat Commun 11:5178. doi:10.1038/s41467-020-18936-1 33057066 PMC7560704

[26] Shue B, Chiramel AI, Cerikan B, To T-H, Frölich S, Pederson SM, Kirby EN, Eyre NS, Bartenschlager RFW, Best SM, Beard MR. 2021. Genome-wide CRISPR screen identifies RACK1 as a critical host factor for flavivirus replication. J Virol 95:e0059621. doi:10.1128/JVI.00596-21 34586867 PMC8610583

[27] Hoffmann H-H, Schneider WM, Rozen-Gagnon K, Miles LA, Schuster F, Razooky B, Jacobson E, Wu X, Yi S, Rudin CM, MacDonald MR, McMullan LK, Poirier JT, Rice CM. 2021. TMEM41B is a pan-flavivirus host factor. Cell 184:133–148. doi:10.1016/j.cell.2020.12.005 33338421 PMC7954666

[28] Luu AP, Yao Z, Ramachandran S, Azzopardi SA, Miles LA, Schneider WM, Hoffmann H-H, Bozzacco L, Garcia G, Gong D, Damoiseaux R, Tang H, Morizono K, Rudin CM, Sun R, Arumugaswami V, Poirier JT, MacDonald MR, Rice CM, Li MMH. 2021. A CRISPR activation screen identifies an atypical rho GTPase that enhances Zika viral entry. Viruses 13:2113. doi:10.3390/v13112113 34834920 PMC8623001

[29] Ma H, Dang Y, Wu Y, Jia G, Anaya E, Zhang J, Abraham S, Choi J-G, Shi G, Qi L, Manjunath N, Wu H. 2015. A CRISPR-based screen identifies genes essential for west-nile-virus-induced cell death. Cell Rep 12:673–683. doi:10.1016/j.celrep.2015.06.049 26190106 PMC4559080

[30] Lin DL, Inoue T, Chen Y-J, Chang A, Tsai B, Tai AW. 2019. The ER membrane protein complex promotes biogenesis of dengue and Zika virus non-structural multi-pass transmembrane proteins to support infection. Cell Rep 27:1666–1674. doi:10.1016/j.celrep.2019.04.051 31067454 PMC6521869

[31] Abell BM, Jung M, Oliver JD, Knight BC, Tyedmers J, Zimmermann R, High S. 2003. Tail-anchored and signal-anchored proteins utilize overlapping pathways during membrane insertion. J Biol Chem 278:5669–5678. doi:10.1074/jbc.M209968200 12464599

[32] Chung Y, Yim C, Pereira GP, Son S, Kjølbye LR, Mazurkiewicz LE, Weeks AM, Förster F, von Heijne G, Souza PCT, Kim H. 2024. Spc2 modulates substrate- and cleavage site-selection in the yeast signal peptidase complex. J Cell Biol 223:e202211035. doi:10.1083/jcb.202211035 39565596 PMC11579918

[33] Chen Z, Wang C, Feng X, Nie L, Tang M, Zhang H, Xiong Y, Swisher SK, Srivastava M, Chen J. 2021. Interactomes of SARS-CoV-2 and human coronaviruses reveal host factors potentially affecting pathogenesis. EMBO J 40:e107776. doi:10.15252/embj.2021107776 34232536 PMC8447597

[34] Matthaei A, Joecks S, Frauenstein A, Bruening J, Bankwitz D, Friesland M, Gerold G, Vieyres G, Kaderali L, Meissner F, Pietschmann T. 2024. Landscape of protein-protein interactions during hepatitis C virus assembly and release. Microbiol Spectr 12:e0256222. doi:10.1128/spectrum.02562-22 38230952 PMC10846047

[35] Cerikan B, Goellner S, Neufeldt CJ, Haselmann U, Mulder K, Chatel-Chaix L, Cortese M, Bartenschlager R. 2020. A non-replicative role of the 3’ terminal sequence of the dengue virus genome in membranous replication organelle formation. Cell Rep 32:107859. doi:10.1016/j.celrep.2020.107859 32640225 PMC7351112

[36] Hua RH, Bu ZG. 2011. A monoclonal antibody against PrM/M protein of Japanese Encephalitis Virus. Hybridoma (Larchmt) 30:451–456. doi:10.1089/hyb.2011.0027 22008072

[37] Hua R-H, Li Y-N, Chen Z-S, Liu L-K, Huo H, Wang X-L, Guo L-P, Shen N, Wang J-F, Bu Z-G. 2014. Generation and characterization of a new mammalian cell line continuously expressing virus-like particles of Japanese Encephalitis Virus for a subunit vaccine candidate. BMC Biotechnol 14:62. doi:10.1186/1472-6750-14-62 25011456 PMC4094896

[38] Hua RH, Liu LK, Chen ZS, Li YN, Bu ZG. 2013. Comprehensive mapping antigenic epitopes of NS1 protein of Japanese Encephalitis Virus with monoclonal antibodies. PLoS One 8:e67553. doi:10.1371/journal.pone.0067553 23825668 PMC3688998

[39] Welsch S, Miller S, Romero-Brey I, Merz A, Bleck CKE, Walther P, Fuller SD, Antony C, Krijnse-Locker J, Bartenschlager R. 2009. Composition and three-dimensional architecture of the dengue virus replication and assembly sites. Cell Host & Microbe 5:365–375. doi:10.1016/j.chom.2009.03.007 19380115 PMC7103389

