# Supplementary material for: SPCS2 serves as a critical host factor for JEV replication by regulating viral protein stability and virion assembly
Source: Microbiol Spectr. 2026 Mar 30;14(5):e03848-25. doi: 10.1128/spectrum.03848-25 (PMC13141937; doi:10.1128/spectrum.03848-25)
Supplement: Supplemental material — Fig. S1 to S3; Table S1. [file spectrum.03848-25-s0001.docx]

**Supplementary Data**

**SPCS2 Serves as a Critical Host Factor for JEV Replication by Regulating Viral Protein Stability and Virion Assembly**

Bei Niu^1*^, Shi-Meng Liu^1*^, Shu-Jian Zhang^1^, Yu-Ting Huang^1^, Jian-Hui Zhang^1^, Sen Hu^1^, Zhi-Gao Bu^1,2#^, Rong-Hong Hua^1,#^

^1^ State Key Laboratory of Animal Disease Control and Prevention, Harbin Veterinary Research Institute, Chinese Academy of Agricultural Sciences, Harbin 150069, China.

^2^ Jiangsu Co-innovation Centre for Prevention and Control of Important Animal Infectious Disease and Zoonoses, Yangzhou University, Yangzhou 225009, China.

# Correspondence author:

Rong-Hong Hua, [huaronghong@163.com](mailto:huaronghong@163.com), ORCID: 0000-0001-7034-5766

Zhi-Gao Bu, [buzhigao@caas.cn](mailto:buzhigao@caas.cn), ORCID: 0000-0001-9242-4211

* Bei Niu and Shi-Meng Liu have contributed equally to this study.

**
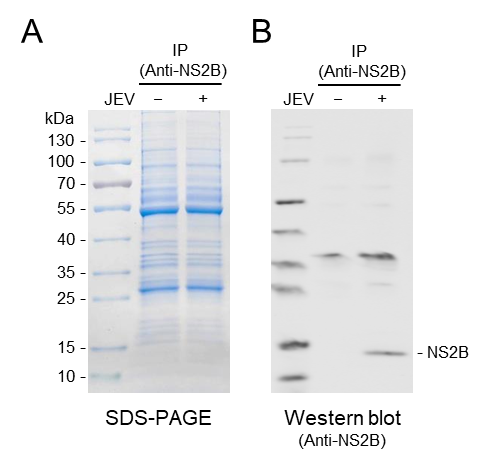
**

**Fig. S1 SDS-PAGE and western blot analysis of immunoprecipitated proteins.** The lysates of JEV-infected or uninfected cells were subjected to immunoprecipitation using JEV NS2B-specific antibodies. Immunoprecipitated proteins were analyzed using SDS-PAGE (A) and western blotting (B).

**
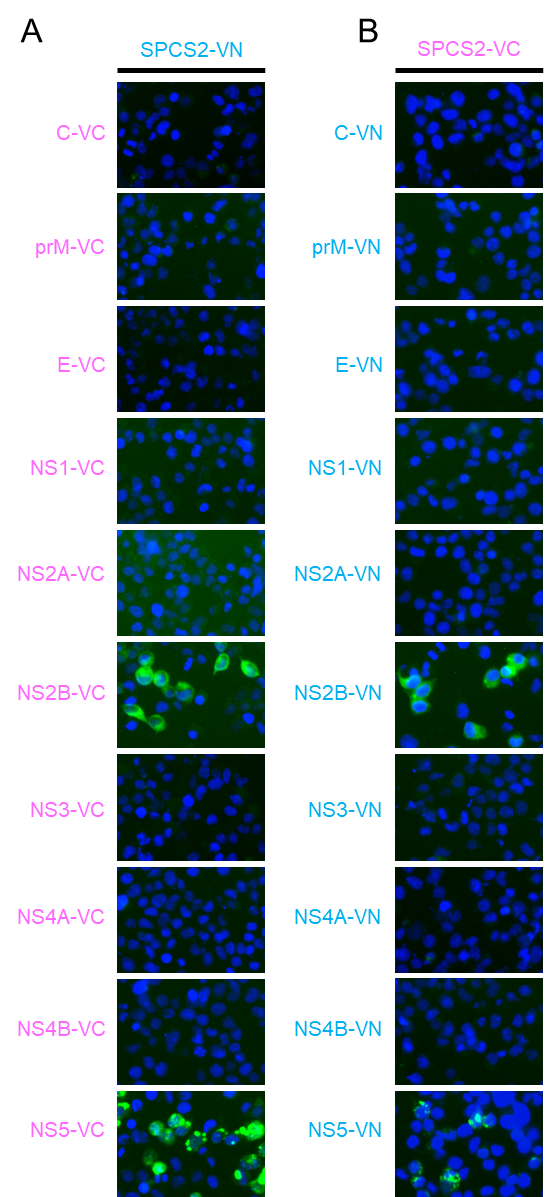
**

**Fig. S2 BiFC screening of the interaction between SPCS2 and JEV proteins.** To screen for the interaction between SPCS2 and JEV proteins, SPCS2-VN and the indicated -VC tagged viral protein (A) or SPCS2-VC and the indicated -VN tagged viral proteins were co-expressed in HEK-293 cells by plasmid transfection. At 12 h post-transfection, the cell nuclei were stained with Hoechst 33342, and the cells were visualized and photographed using a fluorescence microscope.

**
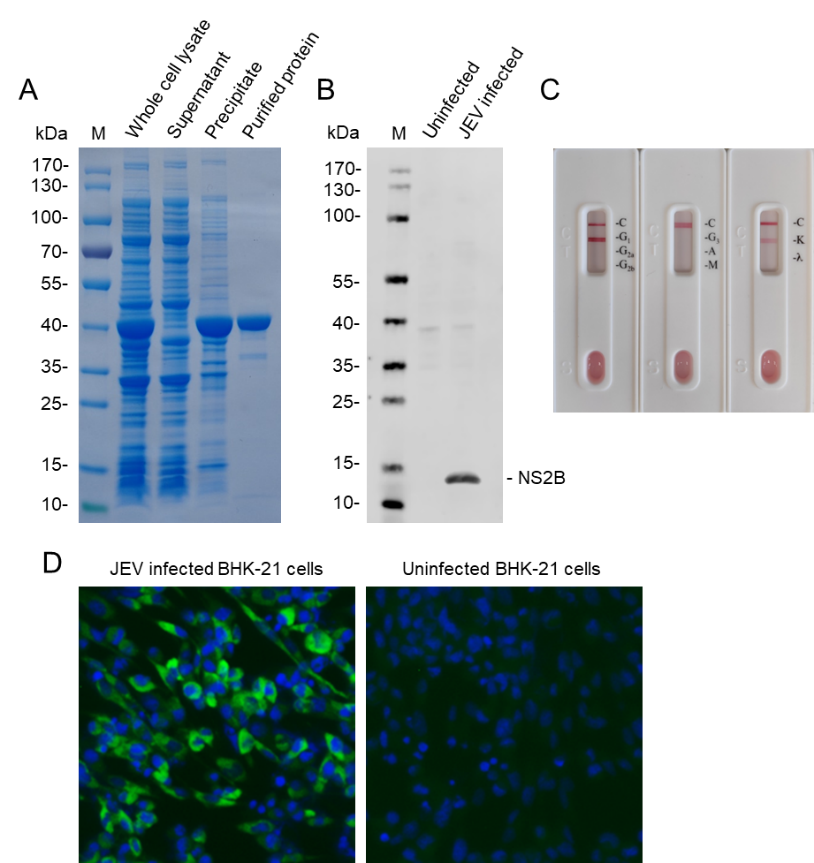
**

**Fig. S3 Preparation and identification of JEV NS2B specific monoclonal antibody.** To generate JEV NS2B specific monoclonal antibody (mAbs), NS2B protein was expressed as a GST-fused protein and purified by gel extraction (A). Mice were immunized with purified protein emulsified with an adjuvant. After cell fusion and ELISA testing, a positive cell clone was screened and designated as 14A3. mAb 14A3 recognized the JEV NS2B protein in JEV-infected cells by western blotting (B). The isotype of mAb 14A3 was tested as IgG1, and the light chain of mAb 14A3 was identified as kappa (C). mAb 14A3 recognized the native NS2B protein in JEV-infected cells by indirect immunofluorescence assay (D).

**Table S1. List of plasmids used in this study.**

| **Plasmid** | **Source** |
| --- | --- |
| pWNVrepdCME-GFP | (1) |
| pCAG-J-CME | (2) |
| pCAG-J-NS2B-FLAG | (2) |
| pCAG-J-NS5-FLAG | (2) |
| pSpCas9-BB-2A-GFP (PX458) | Addgene, #48138 |
| pSpCas9-BB-2A-GFP-SPCS2 | This study |
| pCAG-SPCS2-His | This study |
| pCAG-SPCS2-VN | This study |
| pCAG-SPCS2-VC | This study |
| pCAG-SPCS2 | This study |
| pCAG-J-C-VN | (2) |
| pCAG-J-prM-VN | (2) |
| pCAG-J-E-VN | (2) |
| pCAG-J-NS1- N | (2) |
| pCAG-J-NS2A- N | (2) |
| pCAG-J-NS2B-VN | (2) |
| pCAG-J-NS3-VN | (2) |
| pCAG-J-NS4A-VN | (2) |
| pCAG-J-NS4B-VN | (2) |
| pCAG-J-NS5-VN | (2) |
| pCAG-J-C-VC | (2) |
| pCAG-J-prM-VC | (2) |
| pCAG-J-E-VC | (2) |
| pCAG-J-NS1-VC | (2) |
| pCAG-J-NS2A-VC | (2) |
| pCAG-J-NS2B-VC | (2) |
| pCAG-J-NS3-VC | (2) |
| pCAG-J-NS4A-VC | (2) |
| pCAG-J-NS4B-VC | (2) |
| pCAG-J-NS5-VC | (2) |

**References**

1. Li W, Ma L, Guo L-P, Wang X-L, Zhang J-W, Bu Z-G, Hua R-H. 2017. West Nile virus infectious replicon particles generated using a packaging-restricted cell line is a safe reporter system. Scientific Reports 7:3286.

2. Ma L, Li F, Zhang J-W, Li W, Zhao D-M, Wang H, Hua R-H, Bu Z-G. 2018. Host Factor Spcs1 Regulates the Replication of Japanese Encephalitis Virus Through Interactions with Transmembrane Domains of Ns2b. Journal of Virology 92:e00197-18.
